# The impact of a heat therapy intervention on pain and fibromyalgia symptoms in patients with fibromyalgia: a pilot study

**DOI:** 10.3389/fpain.2025.1526491

**Published:** 2025-03-13

**Authors:** Andrea L. Chadwick, Chloe Shi, Miranda McMillan, Josh Miller, Jinxiang Hu, Paige C. Geiger

**Affiliations:** ^1^ Department of Anesthesiology, Pain, and Perioperative Medicine, University of Kansas School of Medicine, Kansas City, KS, United States; ^2^ School of Medicine, University of Missouri, Kansas City, MO, United States; ^3^ Department of Obstetrics and Gynecology, Wayne State University, Detroit, MI, United States; ^4^ Department of Biostatistics and Data Science, University of Kansas School of Medicine, Kansas City, KS, United States; ^5^ Department of Cell Biology and Physiology, University of Kansas School of Medicine, Kansas City, KS, United States

**Keywords:** fibromyalgia, chronic pain, non-pharmacologic intervention, nociplastic pain, heat therapy

## Abstract

**Introduction:**

FM is characterized by widespread musculoskeletal pain and associated somatic symptoms including fatigue, cognitive difficulties, and problems with sleeping. Multidisciplinary treatment of fibromyalgia including pharmacologic and non-pharmacologic interventions are recommended to improve symptoms and physical functioning. The goal of the present pilot investigation was to evaluate the effects of heat therapy via hot water immersion on clinical and objective pain measures in addition to blood measurements of heat shock proteins (HSPs) and inflammatory markers in patients with FM.

**Methods:**

After screening, informed consent, and enrollment into the study, all subjects underwent a baseline pre-intervention evaluation which included a battery of pain phenotyping questionnaires, quantitative sensory testing, and collection of blood for measurements of HSPs and inflammatory markers. Subjects received heat therapy three times a week for four weeks, where they were immersed in hot water for 45 min. After four weeks, participants completed the same battery of testing done at baseline.

**Results:**

We found that four weeks of heat therapy via hot water immersion in patients with FM showed statistically significant reductions in average and worst pain NRS severity scores when compared to baseline. There was also statistically significant improvement in overall impact of fibromyalgia symptoms, physical function, and sleep-related impairment. Regarding heat shock proteins, there was a statistically significant reduction in HSP90 and induction of HSP40 and HSC70. The number of extracellular vesicles were also statistically significantly increased. There were no statistically significant changes found in depression, anxiety, quantitative sensory testing measures, or pro- or anti-inflammatory markers.

**Conclusions:**

As a whole, these findings suggest that heat therapy via hot water immersion may be an effective non-pharmaceutical intervention for patients with FM and that its analgesic benefits may be related to decreases in HSP 90 and increases in HSP 40 and 72. Further large-scale, well-powered studies are needed to confirm our preliminary clinical and translational results.

## Introduction

Fibromyalgia (FM) is a chronic pain condition that is present in 2%–8% of the international population (1) with a higher prevalence in in women, with male:female ratios that have been reported to range from 1:2 to 1:9 (2–5). FM is characterized by widespread musculoskeletal pain and associated somatic symptoms including fatigue, cognitive difficulties, and problems with sleeping (4, 6–8). Patients with FM have been shown to have abnormal physiologic pain processing parameters including reduced pain thresholds consistent with hyperalgesia and allodynia and decreased endogenous pain modulation as measured by conditioned pain modulation (9–16). The pathophysiology of FM has been shown have a variety of central nervous system mechanisms including amplified nociceptive signaling in addition to decreased endogenous inhibition of pain (1, 17). It has been shown that an imbalance of pro-inflammatory and anti-inflammatory cytokines is a main contributor to chronic central nervous system sensitization and amplified pain processing (18, 19). As such, increased serum levels of pro-inflammatory cytokines has been described in patients with FM (20–24).

Due to the complexity of FM, there is no standard intervention for reducing pain, improving FM symptoms, and improving quality of life in FM patients. Guidelines for the treatment of FM have been published by international societies and working groups and highlight the importance of multidisciplinary treatment strategies (25–34). While pharmacologic therapy using both on-label and off-label medications are routinely used in practice to treat FM, there is a general lack of moderate to strong research-based evidence for the use of these pharmaceutical interventions and many of them have undesirable side effects that limit their use. Exercise and physical rehabilitation interventions have been investigated in FM patients and have shown excellent efficacy in reducing pain and fatigue and improving physical functioning and global quality of life in numerous studies (35–39). Although there is ample evidence that exercise and physical rehabilitation are excellent treatments for FM, exercise and physical rehabilitation can be difficult for some patients with severe pain or physical deconditioning from sedentary behavior. Despite the known benefits of exercise for FM, many patients have difficult initiating or maintaining this treatment regimen due to fear avoidance of pain (40) and rebound hyperalgesia (41). Thus, there exists is a need for a passive, non-pharmaceutical intervention that could help to reduce pain, improve physical functioning, and lead to a better quality of life.

Saunas and hot baths have been used throughout history as a way of improving quality of life and well-being (42) and have been associated with benefits to the cardiovascular system (43). Heat shock proteins (HSPs) are a family of molecular chaperones that are the main effectors of the benefits of heat therapy. There are several stimuli that result in the upregulation in HSP expression such as hypoxia, decreased pH, oxidative stress, exercise, and exposure to heat (44, 45). Two HSPs that have been reported to be involved in pain and pain-related mechanisms are HSP72 and HSP90. HSP72 has been shown to have a neuroprotective role in attenuating neuropathic pain in rats after peripheral nerve injury (46) and in delaying the development of hyperalgesia and allodynia in rats with painful diabetic neuropathy (47). HSP90 induces the production of proinflammatory cytokines through the activation of the Toll-like receptor 4 (TLR4) inflammatory pathway (48), while its inhibition can attenuate the inflammatory response (49). Given this knowledge, investigators have performed research on whether heat therapy can improve symptoms of pain and function in FM patients and while only a few studies exist, they show some promise (50–53). None of the published studies using heat thearpy for FM have specifically examined the use of hot water immersion (via soaking in a hot therapy bath) as a stand-alone heat therapy intervention.

The goal of the present pilot investigation was to evaluate the effects of heat therapy on clinical and objective pain measures, HSPs, and inflammatory markers in patients with FM. Given the basic science benefits and results from the prior research, we hypothesized that heat therapy via hot water immersion would lead to an changes in HSP expression and decreases in pro-inflammatory markers, which would then be associated with a decrease in subjective and objective pain outcomes.

## Methods

### Ethical approval

Prior to patient enrollment, all study procedures and experimental protocols were approved by the Institutional Review Board at the University of Kansas Medical Center. This study was registered as a clinical trial at clinicaltrials.gov. Identifier: NCT03768947 prior to patient recruitment and enrollment. All patient recruitment, enrollment, and study procedures took place at the University of Kansas Medical Center from August 2019 to March 2020, when study procedures had to cease due to the COVID-19 pandemic. All subjects provided oral and written informed consent prior to participation in the study, as set forth by the *Declaration of Helsinki*.

### Overall study design

This study was designed as a non-randomized observational clinical trial with a single intervention of interest (heat therapy via hot water immersion). After screening, informed consent, and enrollment into the study, all subjects underwent a baseline pre-intervention evaluation which included a battery of pain phenotyping questionnaires that assessed symptoms including pain severity, physical function, depression, anxiety, and sleep. A battery of Quantitative Sensory Testing (QST) was also performed at this baseline pre-intervention visit. Within 30 days of pre-intervention testing, the subjects began the one month heat therapy intervention protocol. Within 48 h of the completion of the 1-month heat therapy intervention, subjects underwent a post-intervention evaluation which included the same battery of pain phenotyping questionnaires and QST. Blood samples were collected by venipuncture at the baseline pre-intervention and post-intervention visits for analysis of pro- and anti-inflammatory markers and HSP levels.

### Inclusion and exclusion criteria

Participants were eligible for inclusion if they were adults between 18 and 65 years old who reported sedentary lifestyles. Participants required to have a of diagnosis of fibromyalgia and were identified as meeting criteria for diagnosis through the American College of Rheumatology 2016 FM Diagnostic Criteria (54). Participants were required to have an average Brief Pain Inventory (BPI) visual numeric pain score greater than 4 and were required to have stable dosages of over the counter and prescription medications for at least 30 days prior to screening. Participants were included if they agreed to remain on the same medication regimen throughout the study duration. Participants were excluded if they had any clinically significant cardiovascular or respiratory conditions that would prevent safe hot water immersion.

### Heat therapy intervention

Subjects received heat therapy three times a week for four weeks. Subjects were immersed up to the shoulder in a 40.5°C water therapy bath until rectal temperature (T_re_) reached 38.5°C, which was generally achieved in patients in approximately 30 min. Subjects then remained in the water therapy bath submerged to waist level to maintain T_re_ between 38.5°C and 39.0°C for another 15 min (total of 45 min in the water therapy bath). Following hot water immersion, subjects exited the water therapy bath and were monitored for another 10 min, or until T_re_ fell below 38.5°C. This protocol was adapted from heat therapy intervention done via hot water immersion from the Minson Lab (55).

Core temperature was monitored using a sterile rectal thermistor probe (401 A/C, Advanced Industrial Systems, Inc., Harrods Creek, KY) and was self-inserted by the participants to ∼10 centimeters (cm) past the anal sphincter. Heart rate and blood pressure measurements were taken every 10 min during the study visit to ensure adequate hydration and circulation. Subjects were allowed to drink water *ad libitum* during the hot water immersion. Body weight was measured before and immediately after all sessions to ensure adequate hydration. Subjects that did not drink enough water to compensate for sweat loss (body weight loss >1%) drank additional fluids to make up this difference prior to leaving the research facility.

### Pain phenotyping questionnaires

The Brief Pain Inventory (BPI) is an 11-item validated questionnaire used to assess two different aspects of pain: pain severity and pain interference using a scale of 0–10 with 0 being no pain and 10 being as bad as one can imagine (56). In this study, average pain visual numerical score (VNS) over the past two weeks and worst pain VNS in the last two weeks were of particular interest. This survey was administered to participants to ascertain their subjective level of pain severity at baseline (pre-intervention) and again at 24–48 h post-intervention to determine their level of pain severity once heat therapy had been completed. This is the primary subjective outcome measure for assessing pain in this study.

The Fibromyalgia Impact Questionnaire (FIQ-R) is a specific and validated measure of fibromyalgia (FM) symptoms of FM patients. This survey is used to determine how FM symptoms affect different aspects of a patient's life such as sleep, ability to perform household tasks, and depression (57). FIQ-R consists of 21 questions and was scored on a scale from 0 to 10 with 10 representing “worst”. FIQ-R is divided into 3 sections: function (9 questions), overall impact (2 questions), and symptoms (10 questions).

Fibromyalgianess (FMness) is a measure of pain and co-morbid symptom extensiveness and severity. It was calculated by combining the scores from the Widespread Pain Index with the Symptom Severity Scale from the 2016 FM Survey (54, 58) to derive a continuous metric purportedly indicative of the degree of centralized pain present in a given individual (59).

The Patient-Reported Outcomes Measurement Information System (PROMIS) tools have previously been determined to be a valid and reliable way to measure additional pain-related factors associated with FM with a high correlation to FIQ-R (*r* = −.72) (60). PROMIS surveys for physical function, depression, anxiety, and sleep related impairment were administered in this study. PROMIS scores were calculated by taking the raw score and finding the *T*-score which can be found at http://www.healthmeasures.net/explore-measurement-systems/promis.

### Quantitative sensory testing (QST)

Pain testing was performed using a variety of validated tools and instruments, including the Multimodal Automated Sensory Testing (MAST) System, a computerized QST device developed at the University of Michigan, and currently being employed in several clinical trials, including the National Institutes of Health (NIH) Multidisciplinary Approach to the Study of Chronic Pelvic Pain (MAPP) Network (61).

#### Generalized pressure pain sensitivity – thumbnail

Thumbnail pressure was applied using the MAST System (62) - an automated QST platform featuring a control computer, a touch screen for patient feedback, and wireless pressure actuator. Forces were delivered by a 1-cm^2^ rubber probe driven by a miniature servo-motor and housed within a urethane pistol-grip-style case. The MAST delivered an ascending series of discrete pressures (5-s duration; 4 kg/cm^2^/s ramp rate) at 20-s intervals, beginning at 0.25 kg/cm^2^ and increasing in 0.25–0.50 kg/cm^2^ steps. Pain intensity was rated after each stimulus on a 0–100 numerical rating scale (NRS) displayed on the patient's screen (0 = no pain; 100 = most intense pain imaginable). The test was terminated when subjects reach their tolerance or 10 kg/cm^2^. A regression model was used to fit the stimulus-response data and interpolate *Pain 30, Pain40, and Pain50*, defined as the pressure intensity that evokes a mild or moderate level of pain (i.e., 30/100, 40/100, or 50/100). Other variables that were derived include: (1) thumb pressure pain threshold (*thumb-PPT*), defined as the first thumb pressure in a series of at least two consecutive thumb pressures that elicited a pain rating >0, and (2) *Pressure Pain Tolerance (thumb-TOL)*, defined as the highest pressure tolerated. This parameter was assessed by applying discrete pressure stimuli to the thumbnail bed.

#### Conditioned pain modulation (CPM)

CPM assessment required a painful “conditioning stimulus” to induce descending anti-nociception and alter pain perception, and a painful “test stimulus” to evaluate the anti-nociceptive response to the conditioning stimulus. CPM was evaluated using two MAST pressure actuators for the test stimulus (non-dominant thumbnail) and the conditioning stimulus (dominant thumbnail). Painful pressure (Pain40, 30-s duration) delivered to the non-dominant thumbnail served as the test stimulus and rated at 10-, 20-, and 30-s on a 0–100 numerical rating scale. Non-painful pressure stimulus (0.25 kg/cm^2^) or moderately painful pressure stimulus (Pain30) delivered to the dominant thumbnail by a second MAST pressure actuator for 1-min served as a neutral and a painful conditioning stimulus, respectively. Parallel to the last 30-s of CPM conditioning, the test stimulus was reapplied to the non-dominant thumbnail for 30-s and the patients were again asked to rate the intensity of the test stimulus three times. CPM magnitude was calculated as the difference (post-pre) in the mean of the three pain ratings given to the test stimulus prior to the conditioning stimuli and the three pain ratings of the test stimulus given during the conditioning stimuli. A negative CPM magnitude value implies functional (inhibitory) CPM and a positive CPM magnitude value implies dysfunctional (facilitatory) CPM.

### Blood sample collection and preparation

25 ml of blood were collected from participants at both the baseline pre-intervention and the post-intervention time points. 8 ml were collected in K_2_EDTA tubes (BD, Franklin Lakes, NJ) and immediately centrifuged at 1,500 × g for 10 min at 4°C. After centrifugation, plasma was carefully aliquoted and then stored at −80°C. The other 17 ml of blood was collected in two 8.5 ml Serum Separator Tubes (BD, Franklin Lakes, NJ) and allowed to clot for 30 min at room temperature. Samples were then centrifuged at 22°C. Serum was aliquoted and stored at −80°C.

### Extracellular vesicles (EVs) isolation

EVs were isolated and purified from serum using qEVsingle SP2 (IZON, qEVsingle SP2) according to the manufacturer instructions. The fractions were further pelleted at 100,000 × g for 1 h. The pellet (exosome fraction) was dissolved in PBS and analyzed by Western Blot or NanoSight. A NanoSight (Nanosight LM10) or a NanoView machine (ExoView® R100) was employed to analyze the particle densities.

### Western blotting procedure

Isolated EVs were heated to 98°C for 5 min in lane marker reducing sample buffer/non-reducing lane marker sample buffer and separated by12%/15% SDS-polyacrylamide gel electrophoresis, followed by transfer onto polyvinylidene fluoride membranes. Membranes were blocked with 5% milk for 1 h, and then incubated in primary antibodies. These antibodies included anti-HSP72 (Enzo, ADI-SPA-810-F), anti-HSP40 (Enzo, ADI-SPA-807-F), anti-HSP25 (Enzo, ADI-SPA-801-F), anti-HSP90 (Novus Biologicals, NBP1-97506) at 4°C overnight. Each specific HRP-conjugated secondary antibody was added accordingly after the membranes were washed in TBST solution five times for 25 min total, and signals were detected by enhanced chemiluminescence.

### Pro- and anti-inflammatory cytokine assay

Serum was analyzed for pro- and anti-inflammatory cytokines using a magnetic multiplex assay (Luminex, Austin, TX). The cytokines measured were Interleukin (IL) -1α, IL-1β, IL-2, IL-3, IL-4, IL-5, IL-8, IL-10, TNF-α, and CX3CL. All reagents, working standards, and samples were prepared as directed. 50 μl of standard, control, or sample were added per well. The diluted Microparticle Cocktail was resuspended by vortexing. 50 μl of the microparticle cocktail was added to each well of the microplate being used. Then the plate was Incubated for 2 h at room temperature on a horizontal orbital microplate shaker. The wells were then washed and 50 μl of the antibody cocktail was added and incubated on a shaker for 1 h at room temperature. Once again, the wells were washed and 50 μl of Streptavidin-PE was added to each well and the plate was incubated at room temperature for 30 min. Finally, the wells were washed and resuspended for analysis.

### Statistical analysis methods

This study was designed as a pilot study to determine feasibility, safety, and compliance of the study protocol. As such, no *a priori* power calculation or sample size was performed. The primary outcome of interest was the change in average visual numerical score (VNS) from the post-heat therapy follow up visit as compared to baseline. Secondary clinical outcomes of interests were the changes in scores from the FIQ-R, FMness, and PROMIS questionnaires as well as QST measures including Pain50, Pain Tolerance (Pain80), and CPM magnitude from the post-heat therapy follow up visit as compared to baseline. Data outcomes of interest are reported in means ± SD. Primary and secondary clinical outcomes of interest measured at baseline and post-heat therapy intervention visits were compared using Wilcoxon signed-rank test. Secondary outcomes pertaining to blood markers included levels of HSPs (HSP 90, 72, 25, 70, and 40), EVs levels, and cytokines including IL-1α, IL-1β, IL-2, IL-3, IL-4, IL-5, IL-8, IL-10, TNF-α, and CX3CL measured at baseline and post-heat therapy intervention visits were compared using paired t-tests.

## Results

### Participants

**Figure 1:**
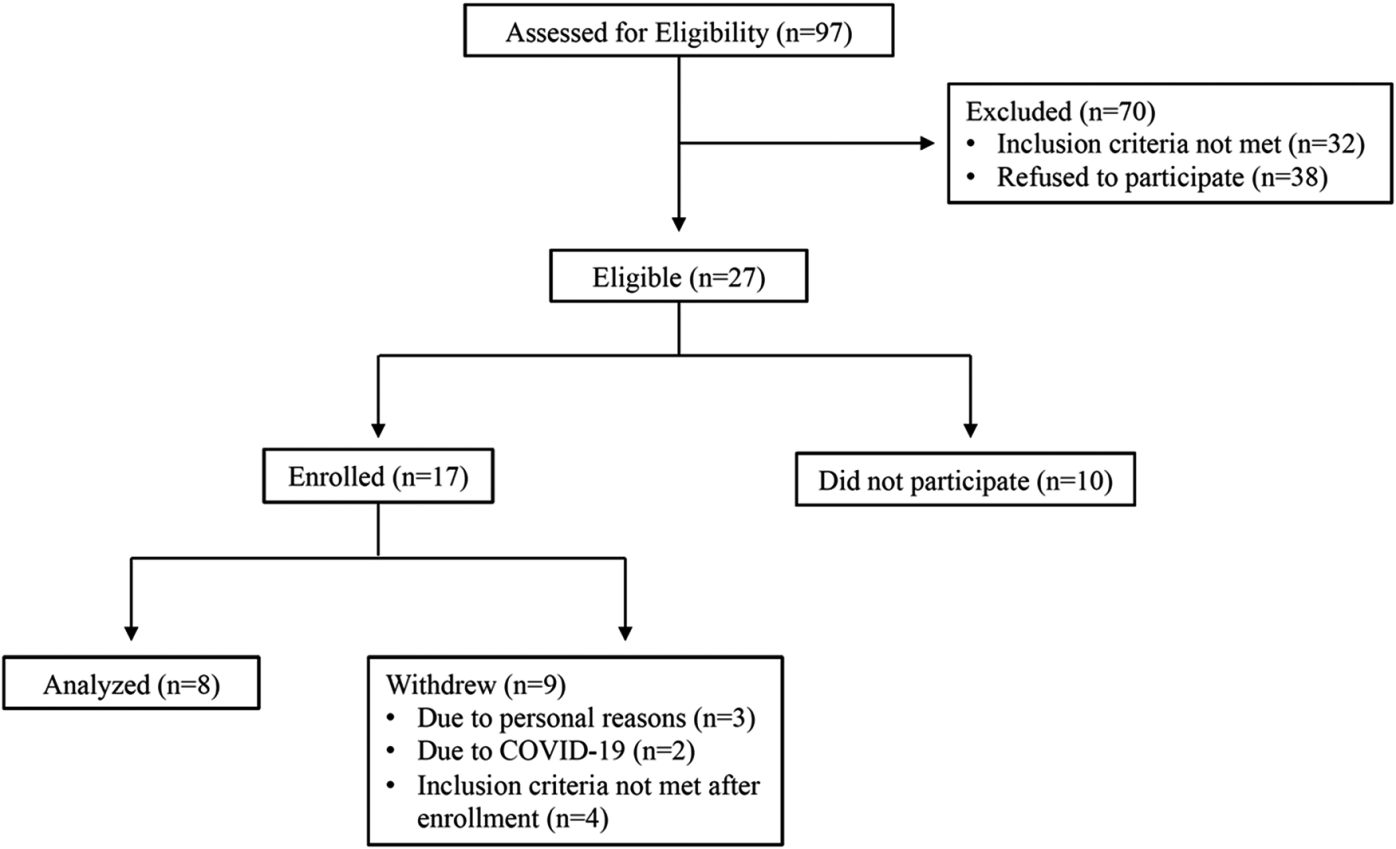
CONSORT diagram.

Out of 62 participants assessed for eligibility, 13 subjects met criteria for enrollment into the study. Please see Figure 1 for CONSORT diagram outlining patient enrollment, exclusion information, and attrition. Demographic characteristics of our all-female cohort are described in Table 1. Data from pain phenotyping questionnaires performed pre-heat therapy intervention and post-heat therapy intervention are reported in Table 2. Out of the 11 participants who initiated the study, only 1 had to withdraw for health issues that were unrelated to the study procedures. Two participants were withdrawn as clinical research was ceased due to the COVID-19 pandemic and crisis. No safety or medical issues arose during any of the study visits for any procedures and all 8 patients who completed the study protocol did so without complication.

**Table 1:** Demographics of the study population.

Variable	Heat therapy intervention group (*n* = 8)
Age (years) [mean ± SD]	45.0 ± 8.12
Weight (kg) [mean ± SD]	106.7 ± 13.7
Body mass index (kg/m^2^) [mean ± SD]	40.0 ± 4.81
Race	
White [*n* (%)]	8 (100)
Ethnicity [*n* (%)]	
Non-Hispanic or Latinx	7 (87.5)
Hispanic or Latinx	1 (12.5)
Relationship status	
Married	5 (62.5)
Never married	1 (12.5)
Divorced	1 (12.5)
Separated	1 (12.5)
Education level [*n* (%)]	
High school/GED	1 (12.5)
Some college	1 (12.5)
Technical/associate’s degree	2 (25)
Bachelor’s degree	2 (25)
Advanced/professional degree	2 (25)

**Table 2:** Pain phenotyping data Pre-heat therapy and post-heat therapy.

Variable	Pre-HT (mean ± SD)	Post-HT (mean ± SD)	Change-HT (mean ± SD)	*p*-value Wilcoxon signed-rank test
FMness	20.375 ± 4.406	19.375 ± 3.068	−1 ± 4.598	.483
BPI average	5.375 ± 1.506	3.750 ± 1.035	−1.625 ± .744	.013
BPI worst	8.250 ± 1.389	6.125 ± 1.808	−2.125 ± 1.642	.035
FIQR	60.896 ± 16.721	36.979 ± 19.612	−23.917 ± 15.031	.008
PROMIS physical function	32.75 ± 5.398	37.737 ± 6.826	4.987 ± 4.386	.014
PROMIS depression	54.962 ± 7.597	50.488 ± 10.951	−4.475 ± 7.706	.108
PROMIS anxiety	58.438 ± 9.926	54.362 ± 12.392	−4.075 ± 9.155	.208
PROMIS sleep related impairment	64.537 ± 5.427	53.700 ± 10.458	−10.838 ± 11.295	.042

FMness, fibromyalgianess; BPI, brief pain inventory; FIQR, fibromyalgia impact questionnaire-revised.

### Effects of heat therapy intervention on subjective pain severity and pain-related indices

**Figure 2:**
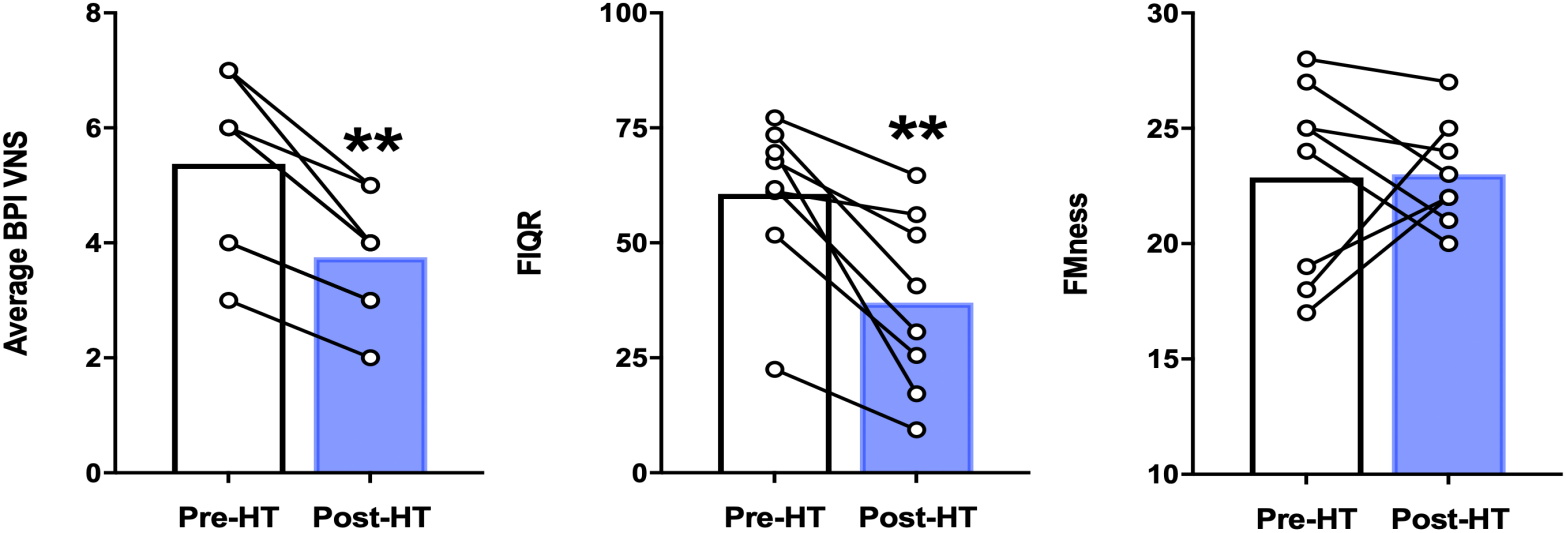
Average BPI pain severity scores, FIQ-R scores, and fMness before and following 4 weeks of heat therapy. 6 subjects underwent 4 weeks of heat therapy (HT) 3 sessions/week of ∼45 min immersed in hot water during with core temperature was increased by 1°C. Data are means ± S.E. **p < 0.05. BPI, brief pain inventory; VNS, visual numerical rating scale; FIQR, fibromyalgia impact questionnaire, FMness, fibromyalgianess.

In our cohort of 8 participants who completed 4 weeks of heat therapy via hot water immersion, average and worst pain severity scores as measured by the BPI showed statistically significant reductions post-heat therapy intervention compared to baseline (*p* = 0.013 and *p* = 0.035, respectively) (Figure 2). In addition, 4 weeks of heat therapy resulted in a statistically significant reduction (*p* = 0.035) in the FIQ-R score (Figure 2). The results are presented as a total score based on a weighted scoring system that is standard for this questionnaire. This reduction in FIQ-R score indicates that the overall impact of fibromyalgia symptoms were reduced for this cohort after heat therapy. FMness was assessed to evaluate the polysymptomatic effects of FM and the varying degrees of centralized pain severity observed in FM patients. While the severity of subjective pain levels and impact of FM symptoms were reduced after 4 weeks of heat therapy in our population, we did not find that the FMness score significantly changed after hot water immersion in this sample of 8 patients (Figure 2).

**Figure 3:**
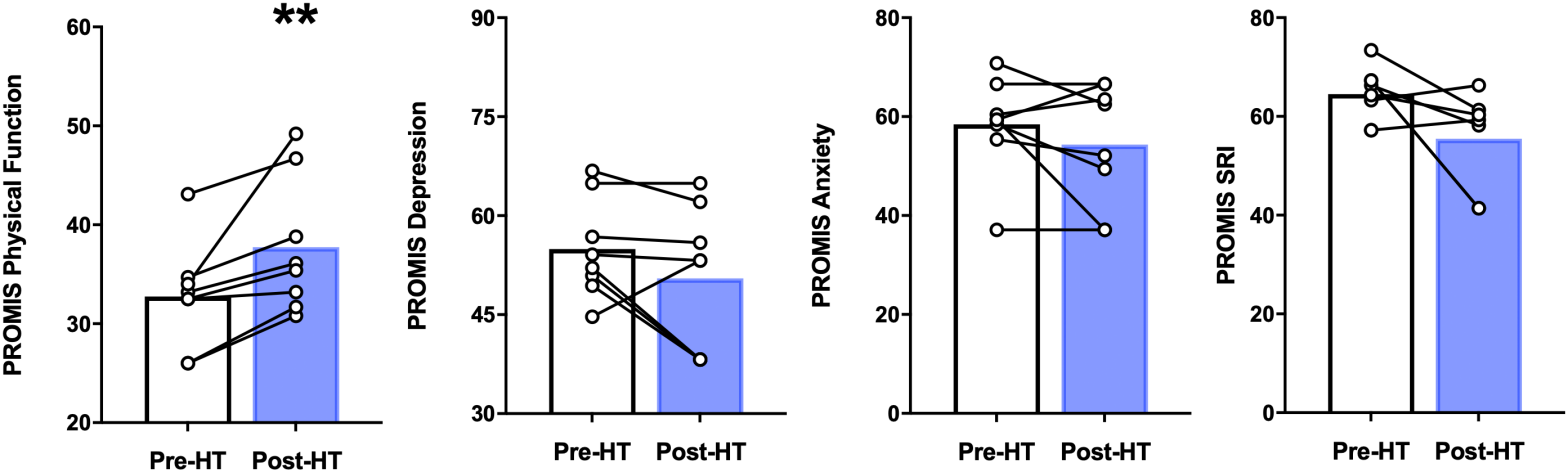
PROMIS physical function, depression, anxiety, and sleep related impairment (SRI) before and after 4 weeks of heat therapy. 8 subjects underwent 4 weeks of heat therapy (HT) 3 sessions/week of 45 min immersed in hot water while core temperature was increased by 1°C. Data are means ± S.E. **p, 0.01.

In our participants, 4 weeks of heat therapy via hot water immersion resulted in a statistically significant (*p* = 0.014) improvement in physical function as assessed by the PROMIS Physical Function 8b questionnaire and in the PROMIS sleep-related impairment (SRI) questionnaire (*p* = 0.042) (Figure 3). 4 weeks of heat therapy did not result in statistically significant changes in PROMIS survey measurements for depression (8a) or anxiety (8a) (Figure 3).

### Effects of chronic heat therapy on physiologic measures of peripheral and central nervous system pain processing

**Figure 4:**
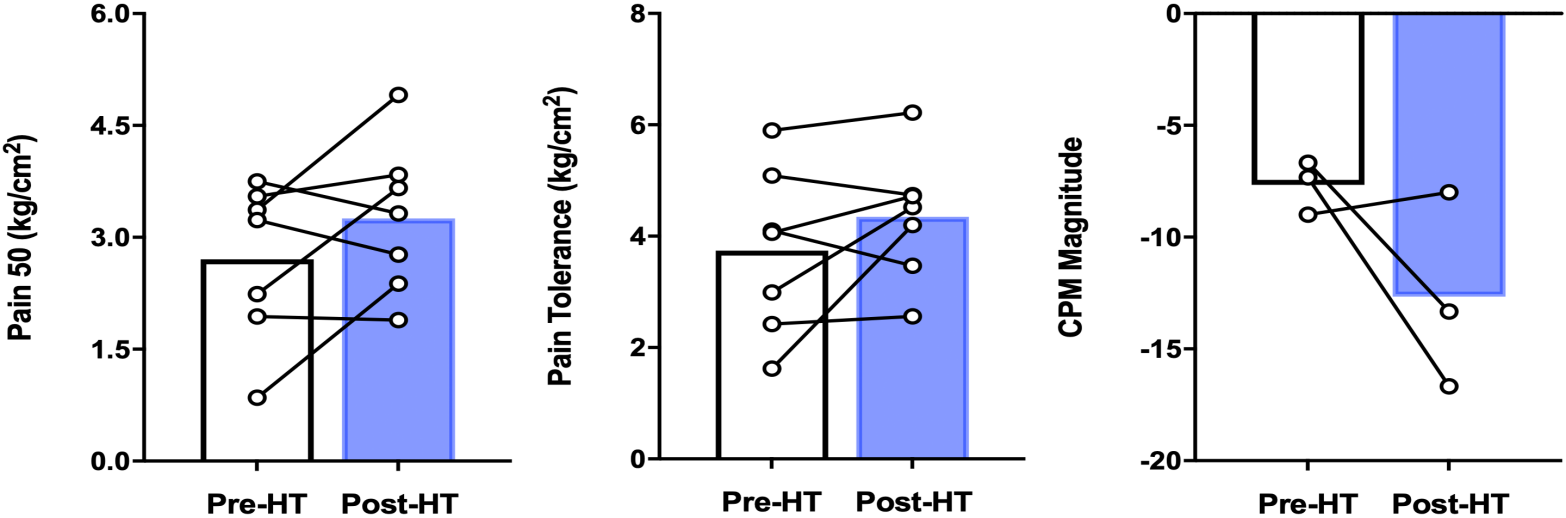
QST parameters in patients with FM before and after 4 weeks of heat therapy. 6 subjects underwent 4 weeks of heat therapy (HT) 3 sessions/week of 45 min immersed in hot water during with core temperature was increased by 1°C. The left panel denotes the pressure needed to elicit a pain score of 50/100, increased, but not significantly from baseline. The middle panel denotes the pressure needed to elicit a pain score of 80/100, again increased but not significantly. The right panel illustrates that pre-heat therapy, FM patients had CPM magnitude consistent with pain facilitation and after heat therapy treatment, had CPM magnitude consistent with pain inhibition. The difference between pre-and post was not statistically significant across all QST parameters. Data are means ± S.E.

A short battery of QST was assessed including pressure pain threshold (PPT) and pressure pain tolerance (PPTol) in addition to CPM using two thumbnail actuators for the conditioning and test stimulus as described earlier. As seen in Figure 4, we found that although not statistically significant, the pressures needed to elicit the pain threshold, numerical pain scores of 30, 40, and 50, and pain tolerance (numerical pain score >80) many participants had pain threshold and tolerance values that trended upwards in these patients.

Pre- and post-heat therapy CPM magnitude was only able to be completed in 3 out of 8 patients due to participants asking to abort the test due to their reported severe pain during the testing protocol. Although not statistically significant, we found that two out of three participant's CPM magnitudes increased in negative magnitude (reflecting improved pain inhibition) after 4 weeks of heat therapy via hot water immersion (Figure 4).

### Effect of heat therapy on inflammatory markers, exosomes, and heat shock proteins

**Figure 5:**
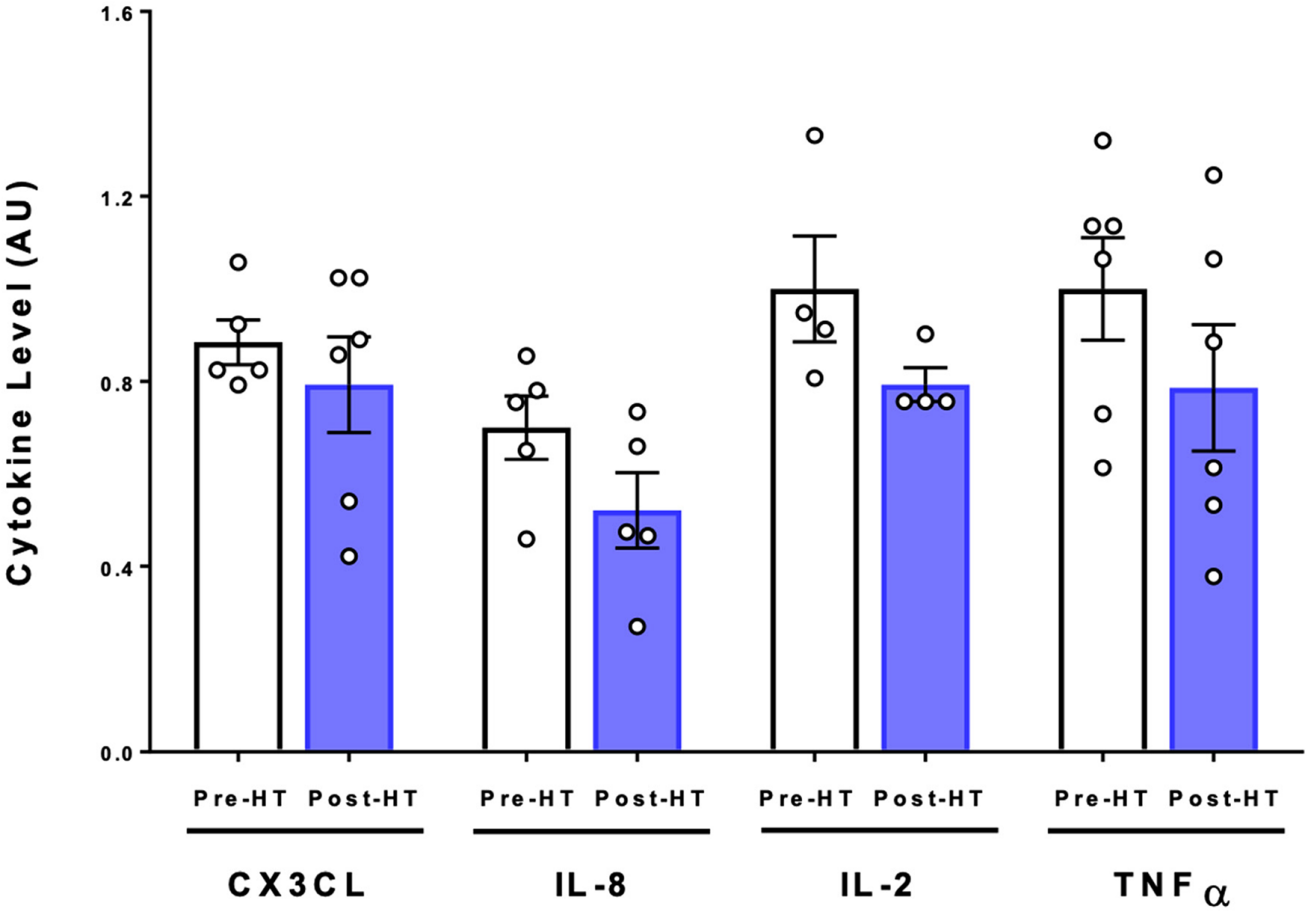
Cytokine expression following 4 weeks of heat therapy. 6 subjects underwent 4 weeks of heat therapy, 3 sessions/week of 45 min immersed in hot water during with core temperature was increased by 1°C. Serum cytokine protein was quantified using commercial ELISA kits and values are relative to pre-heat baseline level for each subject. Data are means ± S.E. for CX3CL, IL-8, IL-2, and TNFα.

To determine whether pro- or anti-inflammatory cytokines levels were altered with 4 weeks of heat therapy, a multiplex cytokine assay was utilized as previously described for samples collected at baseline and at the post-heat therapy intervention follow up visit. Concentrations of IL-1α, IL-1β, IL-3, IL-4, and IL-5 were too low in both pre- and post-heat therapy samples to be detected in all participants. Heat therapy did not cause any statistically significant changes in the inflammatory cytokines CXCL3, IL-8, IL-2, and TNF-α (Figure 5).

**Figure 6:**
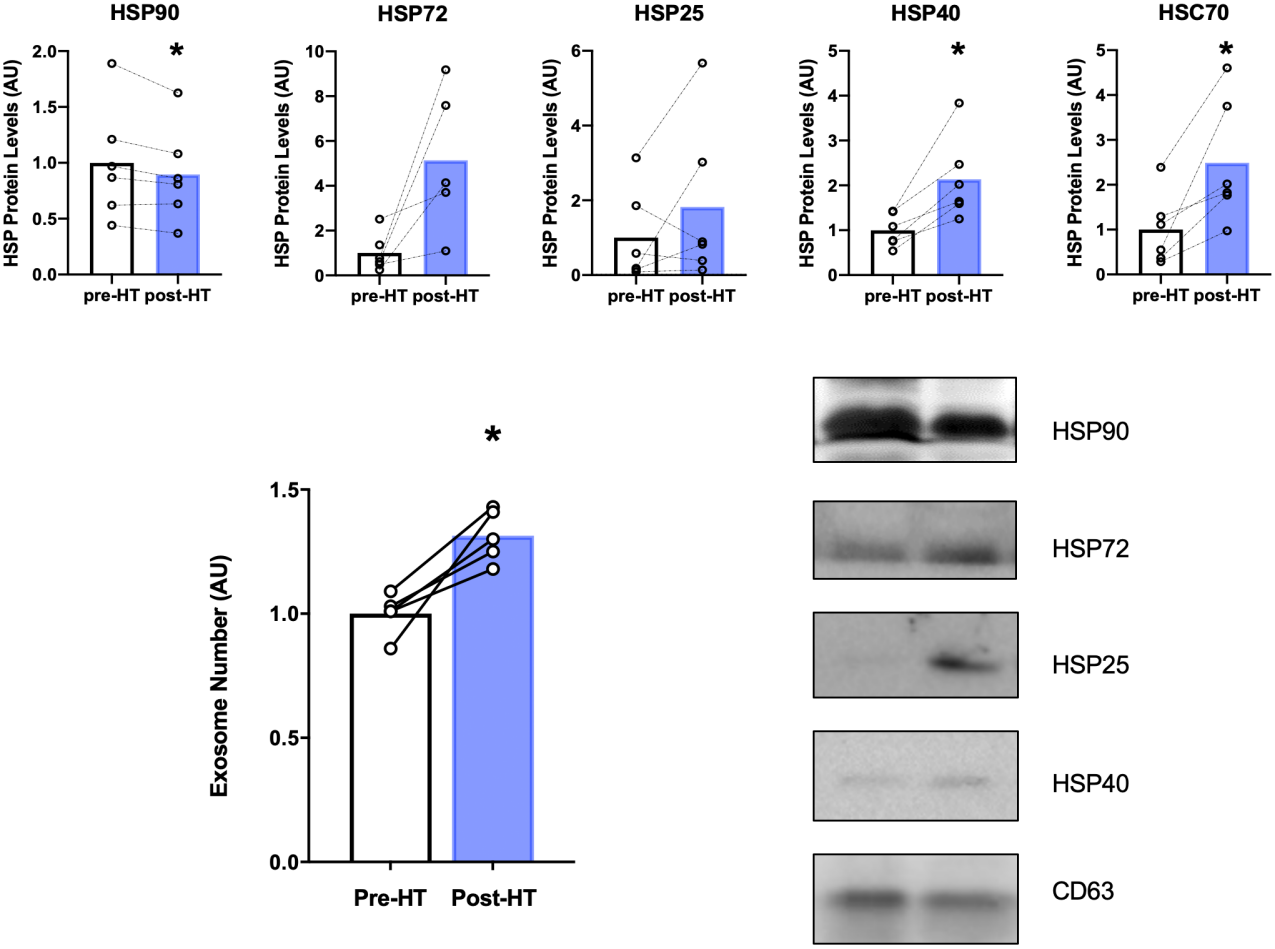
Top panel: HSP protein expression within EVs after 4 weeks of heat therapy. 8 subjects underwent 4 weeks of heat therapy (HT) 3 sessions/week of 45 min immersed in hot water while core temperature was increased by 1°C. Protein expression from isolated EVs was measured by Western blot analyses, expressed relative to pre-heat baseline level for each subject. Data are means ± S.E. *p, 0.05. Lower Left Panel: Serum Extracellular Vesicle number increased following 4 weeks of heat therapy. Serum EV concentration was determined by nanoparticle tracking analysis. Data are means ± S.E. *p, 0.05. Lower Right Panel: Representative Western blots for HSP90, HSP72, HSP25, HSP40, and CD63. Results are shown as pre- and post- 4 weeks of HT.

Heat therapy has been shown in previous research to alter levels of HSPs in the cytosol (63) and in blood-derived EVs (64). We measured HSPs in EVs isolated from plasma due to the potential systemic effect that exosomes exert. As can be seen in Figure 6 there was a statistically significant reduction (*p* = 0.04) in HSP90 within isolated EVs after 4 weeks of heat therapy. HSP72 protein expression increased five-fold on average compared to protein levels in each subject at baseline, but fell just short of statistical significance (*p* = 0.05). HSP25 levels were not significantly increased. Additionally, a statistically significant induction of HSP40 (*p* = 0.01) and HSC70 (*p* = 0.02) were observed after heat therapy. Nanosight results show that EVs as measured by serum exosome number significantly increased following four weeks of heat therapy in patients with FM (*p* < 0.05, Figure 6). The increased number of exosomes may serve as vehicles for the HSPs to exert their effects throughout the body.

## Discussion

We found that four weeks of heat therapy via hot water immersion in patients with FM showed statistically significant reductions in average and worst pain VNS scores when compared to baseline. There was also statistically significant improvement in overall impact of fibromyalgia symptoms, physical function, and sleep-related impairment. Regarding heat shock proteins, there was a statistically significant reduction in HSP90 and induction of HSP40 and HSC70. The number of extracellular vesicles were also statistically significantly increased. There were no statistically significant changes found in depression, anxiety, quantitative sensory testing measures, or pro- or anti-inflammatory markers.

Given there are many negative side effects associated with pharmacologic treatments for FM, it is not surprising that non-pharmacologic interventions such as patient education programs (65, 66), physical activity-based interventions (36–38), and complementary and integrative medicine therapies (67) are increasing in use and popularity amongst clinicians and patients. Heat therapy has been suggested as a potential treatment option in FM and prior studies have been performed showing some benefit. There are a few studies looking at the use of heat therapy as a treatment for pain in patients with FM, however, overall, there is a paucity of data. In a study by Bazzichi et al. (50), 41 FM patients were randomized to either mud bath or balneotherapy (bathing in hot mineral water) and showed that both were efficacious for pain severity, but that mud-bath therapy was superior. Matsumoto et al. (51), performed a study where all participants received a 12-week sauna therapy and water exercise intervention. Short-term (12-weeks) and long-term (6-months) follow up showed a reduction in pain score severity of 28%–77% as well as significant improvements in quality of life measures. Heat-lamp therapy has been used in one study in conjunction with other treatments (massage therapy, aerobic exercise) and while this combination of treatments improved pain in FM patients, it is unclear what precise effect heat therapy had in these outcomes (52). Finally, in a study by Panton et al. (53), laser heat therapy was investigated against a sham therapy in 38 patients with FM and showed that it may improve upper body pain and mobility, but that further studies were warranted given the small sample size. Our results are consistent with these previous studies regarding the effect of heat therapy on pain severity in FM patients. Contributing to the data supporting the use of heat therapy in FM, our study provides a non-pharmacologic alternative to managing chronic pain in FM. However, the specific physiologic explanations for improved pain severity with heat therapy have not been adequately researched.

Hot water immersion has been shown to have broad positive systemic effects (42, 55, 68) and therefore could impact numerous quality of life factors for FM patients including widespread pain, fatigue, and sleep disturbances. The topical action of heat may be another contributing factor in the improvement in pain severity we observed. A study by Michlovitz et al. found heat wrap therapy beneficial in pain relief of different wrist pathologies and this finding was rationalized by the decrease in small nonmyelinated C-fiber activity seen with topical heat therapy that leads to the inhibition of nociceptive spinal cord signals (69).

Heat therapy in the form of hot baths or saunas has been utilized for centuries, with reports of improved quality of life and overall improved well-being (42), however few studies have investigated the potential physiological health benefits. The first comprehensive investigation of the cardiovascular benefits of long-term heat treatment in young, sedentary humans was recently performed (55). Brunt et al., found that 8 weeks of repeated heat therapy by hot water immersion resulted in increased endothelial function (measured via flow-mediated dilation), reduced arterial stiffness, reduced mean arterial and diastolic blood pressure, and reduced carotid intima media thickness. Incredibly, these cardiovascular adaptations were on par with what is typically observed with exercise training in previously sedentary subjects. The benefits of heat therapy are mediated by HSPs, a family of chaperones induced by various acute and chronic conditions such as elevated temperatures, oxidative stress, heavy metals, and exercise. In addition to their well-known chaperone function, HSPs have important cytoprotective and anti-inflammatory responses (70).

HSPs function as part of the heat stress response, a conserved mechanism for the body to mitigate cellular stress and maintain cellular function (71, 72). Not surprisingly, changes in HSP expression profile and cellular localization are linked to numerous disease states. In turn, elevated expression of HSPs have been shown to improve homeostasis in other cells through non-cell autonomous processes (73). HSPs we observed had a change associated with heat therapy were HSP90, HSP40, and HSC70. A decrease in HSP90 is of particular interest in FM patients as HSP90 has been associated with the production of proinflammatory cytokines through the activation of the Toll-like receptor 4 (TLR4) inflammatory pathway (48) and its inhibition alleviated neuropathic pain in preclinical models (49). Additionally, HSP90 has been implicated in the elevation of IL-8 levels with the involvement of TLR4^55^. The significant decrease in HSP90 seen may ultimately lead to a decrease in inflammation via decrease IL-8 and be an important factor contributing the overall decrease in pain. HSP90 inhibition as a method of ameliorating inflammatory pain by the mechanisms described above has been studied by several different groups with consistent findings (74). However, there are no other studies that have been done to describe our specific heat-therapy specific mechanism of reducing HSP90 levels. This finding is an exciting development in the future of chronic pain management. HSP40 and HSC70 have been investigated much less regarding their role in pain. A study done by Iftinca et al. proposed HSC70 mediated inhibition of transient receptor potential vallinoid type 1(TRPV1) may play a protective role in suppressing TRPV1 activated inflammatory pain signals (75). The importance of HSP40 in pain has not yet been explored. Our findings suggest that elevated levels of HSP40 may play an additional role in mediating inhibition of pain signaling and anti-inflammatory signals.

While HSPs may exert their protective and anti-inflammatory effects intracellularly, they can also be secreted into the circulation via EVs. Secreted exosomes and cell surface-released microvesicles are the major EV components and these vesicles effectively travel through the circulation and can be taken up into distant organs and cells. EVs have been shown to act as key regulators of nerve regeneration, synaptic function and behavior (76). In addition, EVs have been shown to be specifically taken up by neurons in which they mediate neuroprotection against cellular stress (77). This protective effect is thought to be due to proteins released from EVs inside the recipient cell. In this manner, post-heat therapy-derived EVs containing increased expression levels of HSPs could effectively decrease inflammation in peripheral nerves. The potential for EVs to act as therapeutic agents has been explored in Alzheimer's Disease (78) and pathological conditions of the central nervous system (76). Our findings from the present study is the first to examine this innovative idea of delivering anti-inflammatory HSPs via EVs to areas of the body susceptible to inflammation and dysfunction in FM patients.

The practical implications of our findings are suggestive of the potential utility of a safe, low-cost, and effective non-pharmacologic intervention to improve pain and the impact of fibromyalgia symptoms as well as physical functioning in patients with FM. Our findings also provide patients with FM and the clinicians who treat them an alternative strategy that can be easily introduced into a comprehensive and multi-disciplinary treatment program. We also believe that in patients who are significantly deconditioned or struggle with initiating or maintaining an exercise or physical rehabilitation program, that heat therapy may possibly be an excellent adjunctive intervention to provide additional benefit and potentially improve adherence.

Although there are numerous strengths to this translational pilot study, there are limitations that deserve mention. First, we acknowledge the small sample size of the present study, which limits formal conclusions regarding non-significant statistical findings that are likely due to low power. However, numerous findings including our primary outcome were found to be statistically significantly improved despite our small sample size. We also acknowledge that there may have been differences in participants who took part in study procedures ahead of the COVID-19 pandemic that are not able to be ascertained due to having to stop study procedures with the pandemic's impact on clinical research. Another limitation is the inability to draw full conclusions on the inflammatory cytokine panels for numerous patients due to indetectable concentrations in serum via multiplex testing. Future studies will likely utilize an *ex-vivo* whole blood lipopolysaccharide stimulation model wherein toll-like receptor 4 global cytokine chemokine release can be scored and measured (79).

## Conclusion

In summary, we found that four weeks of heat therapy via hot water immersion in participants with FM led to statistically significant reductions in average and worst pain NRS severity scores as well as statistically significant improvement in overall impact of fibromyalgia symptoms, physical function, and sleep-related impairment. Analysis of participants blood revealed that four weeks of heat therapy was associated with a statistically significant reduction in HSP90 levels. Furthermore, a statistically significant increase in number of extracellular vesicles and induction of HSP40 and HSC70 blood levels were noted after four weeks of heat therapy in patients with FM. Finally, the number of extracellular vesicles were also statistically significantly increased. As a whole, these findings suggest that heat therapy via hot water immersion may be an effective non-pharmaceutical intervention for patients with FM and that its analgesic benefits may be due to decreases in HSP 90 and increases in HSP 40 and 72 – which although in our study did not show significant changes in inflammatory cytokines, have been associated with improvements in inflammation. Further large-scale, well-powered studies are needed to confirm our preliminary clinical and translational results.

## Data Availability

The raw data supporting the conclusions of this article will be made available by the authors, without undue reservation.
